# Tumor mutational burden as a new biomarker for PD-1 antibody treatment in gastric cancer

**DOI:** 10.1186/s40880-019-0417-1

**Published:** 2019-11-14

**Authors:** Gunnar Folprecht

**Affiliations:** Medical Department I, University Hospital Carl Gustav Carus, 01307 Dresden, Germany

Gastric cancer is the fifth most frequently occurring cancer worldwide. China has the largest gastric cancer population and nearly half of the patients are diagnosed as advanced-stage [1]. The prognosis of most patients with advanced gastric cancer (AGC) remains dismal even despite the approval of several novel chemotherapy drugs and targeted therapy agents. Similarly, immune checkpoint inhibitors (ICIs) such as programmed death-1 (PD-1) antibody have shown promising activity in various solid tumors. Two PD-1 antibodies, pembrolizumab [2] and nivolumab [3], were approved for AGC patients who had treatment failure with chemotherapy. However, the overall response rate (ORR) and the survival benefits of pembrolizumab (11.6%; OS 5.6 months) and nivolumab (nivolumab vs placebo ORR 11% vs 0%, *P* < 0.0001; OS 5.3 vs 4.1 months, *P* < 0.0001) were moderate.

Several biomarkers were found for PD-1 antibodies in AGC such as microsatellite instability-high (MSI-H) and programmed cell death-ligand 1 (PD-L1) expression [4, 5]. According to the Cancer Genome Atlas classification, MSI-H accounted for 22% of the gastric cancers [6]. However, ACG patients with MSI-H only constitutes of a small subgroup in clinical practice. Therefore, a more common biomarker for ICI treatment in AGC is needed. PD-L1 expression was correlated with a higher response to pembrolizumab in patients with AGC who had progressed after at least two prior systemic therapies in the KEYNOTE-059 study. However, pembrolizumab failed to show longer survival than chemotherapy as the second-line in AGC patients with PD-L1 Combined Positive Score (CPS) ≥ 10 in KEYNOTE-061 trial [7]. In addition, responses were observed regardless of the tumors’ PD-L1 status in the CheckMate-032 trial with nivolumab or nivolumab plus ipilimumab [8]. Results from the KEYNOTE-062 showed that even in PD-L1+ (CPS ≥ 1) AGC patients, the first-line response rate of pembrolizumab (14.8%) was quite similar to those from later lines.

Tumor mutational burden (TMB) has been recently described as a new biomarker for PD-(L)1 antibody treatment. However, its predictive effect in AGC had not yet been demonstrated. In a study recently published in Annals of Oncology, Wang et al. [9] have identified TMB as a biomarker for OS benefit in chemo-refractory gastric cancer treated with toripalimab. The authors tested TMB by using whole-exome sequencing (WES) and found that TMB rather than PD-L1 was correlated with a significant survival benefit in AGC. The TMB-High group showed significant superior OS than the TMB-Low group (14.6 vs 4.0 months, HR = 0.48, *P* = 0.038). This study is the first to identify TMB as a predictive biomarker for ICI use in gastrointestinal (GI) tract cancers.

It is also noticeable that TMB-High and PD-L1 positive populations were mostly not overlapping in their study (3.9%). TMB-High and PD-L1 positive patients showed significantly higher ORR (33.3% vs 3.0%) and OS (12.1 vs 4.0 months, HR = 0.47, *P* = 0.027). Only one patient who was PD-L1 negative and was in the TMB-Low group responded to toripalimab. This result suggests that TMB should be further evaluated to identify AGC patients who may respond to PD-1 antibody monotherapy besides PD-L1 testing. For the use in daily clinical practice, a smaller and standardized panel should be developed and needs to be validated in clinical trials.

Further findings in the trial [9] with toripalimab were that the frequency of immune-related adverse events (25.9%) was similar to other trials with pembrolizumab or nivolumab. In contrast, the dramatic responses in Epstein–Barr virus (EBV) positive patients that were observed with six out of six patients achieving a response with pembrolizumab [10] were not reproduced in the current trial where one of the four EBV positive AGC patients showed a response to toripalimab only. Further studies are needed to validate EBV’s predictive effect in AGC.

In summary, TMB may be another good predictive biomarker for PD-1 antibody monotherapy in AGC. A combination of the two biomarkers, TMB-High and PD-L1+, could have the potential to identify a wider range of AGC population who may benefit from ICIs.

## Data Availability

Not applicable.

## Abbreviations

AGC: advanced gastric cancer
ICIs: immune checkpoint inhibitors
PD-1: programmed cell death 1
PD-L1: programmed cell death-ligand 1
ORR: overall response rate
TMB: tumor mutational burden
MSI-H: microsatellite instability high
CPS: Combined Positive Score
WES: whole exome sequencing
GI: gastrointestinal
EBV: Epstein–Barr virus
